# The antidepressant effect of intermittent theta burst stimulation (iTBS): study protocol for a randomized double-blind sham-controlled trial

**DOI:** 10.1186/s13063-023-07674-6

**Published:** 2023-10-02

**Authors:** Marte Christine Ørbo, Ole K. Grønli, Camilla Larsen, Torgil R. Vangberg, Oddgeir Friborg, Zsolt Turi, Matthias Mittner, Gabor Csifcsak, Per M. Aslaksen

**Affiliations:** 1 Department of Psychology, Faculty of Health Sciences, UIT the Arctic University of Norway, Huginbakken 32, Tromsø, N-9037 Norway; 2 Department of Clinical Medicine, Faculty of Health Sciences, UIT the Arctic University of Norway, Tromsø, Norway; 3 Division of Mental Health and Substance Abuse, University Hospital of North Norway, Tromsø, Norway; 4 PET Imaging Center, University Hospital of North Norway, Tromsø, Norway; 5 Department of Neuroanatomy, Institute for Anatomy and Cell Biology, University of Freiburg, Freiburg, Germany; 6 Regional Centre for Eating Disorders, University Hospital of North Norway, Tromsø, Norway

**Keywords:** Intermittent theta burst stimulation, Depression, Cognition, Randomized controlled trial, Magnetic resonance imaging, Genetic factors

## Abstract

**Background:**

Intermittent theta burst stimulation (iTBS) when applied over the left dorsolateral prefrontal cortex (DLPFC) has been shown to be equally effective and safe to treat depression compared to traditional repetitive transcranial magnetic stimulation (rTMS) paradigms. This protocol describes a funded single-centre, double-blind, randomized placebo-controlled, clinical trial to investigate the antidepressive effects of iTBS and factors associated with an antidepressive response.

**Methods:**

In this trial, outpatients (*N* = 96, aged 22–65 years) meeting the diagnostic criteria for at least moderate depression (Montgomery and Aasberg Depression Rating Scale score ≥ 20) will be enrolled prospectively and receive ten, once-a-day sessions of either active iTBS or sham iTBS to the left DLPFC, localized via a neuronavigation system. Participants may have any degree of treatment resistance. Prior to stimulation, participants will undergo a thorough safety screening and a brief diagnostic assessment, genetic analysis of brain-derived neurotropic factor, 5-HTTLPR and 5-HT1A, and cerebral MRI assessments. A selection of neuropsychological tests and questionnaires will be administered prior to stimulation and after ten stimulations. An additional follow-up will be conducted 4 weeks after the last stimulation. The first participant was enrolled on June 4, 2022. Study completion will be in December 2027. The project is approved by the Regional Ethical Committee of Medicine and Health Sciences, Northern Norway, project number 228765. The trial will be conducted according to Good Clinical Practice and published safety guidelines on rTMS treatment.

**Discussion:**

The aims of the present trial are to investigate the antidepressive effect of a 10-session iTBS protocol on moderately depressed outpatients and to explore the factors that can explain the reduction in depressive symptoms after iTBS but also a poorer response to the treatment. In separate, but related work packages, the trial will assess how clinical, cognitive, brain imaging and genetic measures at baseline relate to the variability in the antidepressive effects of iTBS.

**Trial registration:**

ClinicalTrials.gov NCT05516095. Retrospectively registered on August 25, 2022.

**Supplementary Information:**

The online version contains supplementary material available at 10.1186/s13063-023-07674-6.

## Introduction

### Background and rationale

Depression is the leading cause of years lost due to disability in the world, and current drug and psychological treatment approaches are often poorly tolerated or ineffective [1, 2]. High-frequency repetitive transcranial magnetic stimulation (HF rTMS) is now widely accepted by regulatory evidence-based medicine bodies around the world as an effective and safe therapeutic alternative for unipolar depression [3].

Intermittent theta burst stimulation (iTBS) is a modified form of HF rTMS that, when applied over the left dorsolateral prefrontal cortex (DLPFC), has documented antidepressive effects [1, 4–6]. The antidepressant mechanism of action is assumed to be increased neuroplasticity, thus facilitating normalization of neuronal circuit function [7, 8]. iTBS has shown noninferiority compared to rTMS for the treatment of depression, with reduced time consumption and discomfort for the patient [4, 6]. Previous randomized controlled trials (RCTs) have used once-a-day iTBS stimulation with 10 to 30 sessions totally, whereas studies with accelerated iTBS have employed several daily sessions [6, 9, 10]. Still, there is no scientific consensus about the optimal number of treatments or frequency of stimulation needed to achieve symptom reduction in depressed patients. However, the number of published RCT’s is still low [6, 10]. Recent data suggest substantial between patient heterogeneity in the anti-depressant response to iTBS, as is also shown for HF rTMS and other antidepressant treatments [1, 4, 11, 12]. Baseline variables associated with a favourable and relatively fast response to treatment are important to identify, as previous research suggests that early responders are those patients who eventually receive remission [13]. An improved understanding of who might benefit from iTBS or other treatment approaches for depression might be used to refine and improve the effectiveness of iTBS from a research perspective [14–16]. According to previous research, pre-treatment patient characteristics assosciated with a favourable therapeutic effect from HF rTMS are the lesser degree of treatment refractoriness and younger age [13]. Additionally, an antidepressive response observed after 10 sessions is associated with a higher probability of remission in studies using rTMS or iTBS [16]. Baseline cognitive performance could be a significant predictor for the anti-depressive response to iTBS as is previously shown for rTMS [17]. Better performance on executive cognitive tests prior to HF rTMS has been associated with a larger treatment response in major depressive disorder [15]. Improvements in performance on neuropsychological tests have been reported from previous studies applying rTMS or iTBS to the left DLPFC in depressed patients [18–21]. It has been suggested that iTBS may have more beneficial effects on cognition than rTMS in depressed patients [18, 22]. To date, few sham-controlled iTBS studies of depressed patients have included aspects of neurocognition as outcome variables [18]. Given the burden of cognitive dysfunction in MDD, the potential of cognitive improvement from iTBS alongside antidepressant efficacy is an important clinical issue [17].

Studies with rTMS and structural magnetic resonance imaging (MRI) have suggested that reduced volume of the anterior cingulate cortex (ACC) and the amygdala may be pathogenetic factors associated with depression and that smaller volumes and thickness at baseline are associated with better treatment response [18]. It is therefore likely that the same may be observed with iTBS treatment. Reduced white matter integrity measured by fractional anisotropy (FA) is also observed in depressed patients [23], and reduced FA is associated with worse cognitive performance [24]. The microstructure of white matter fibre bundles in the lateral prefrontal regions prior to stimulation may be a predictor of the antidepressive response to iTBS, as previously shown for rTMS [25, 26].

Functional MRI resting state (rs-fMRI) studies have shown that the initial strength of the neural connection between the DLPFC and the ACC could be predictive of the response to iTBS, similar to what is found in studies using rTMS [27, 28]. Furthermore, increased frontal-ACC connectivity is associated with better cognitive performance on executive tasks [29].

The brain-derived neurotrophic factor (BDNF) val66met polymorphism is associated with an antidepressant response in patients with depression [30]. Previous studies using regular rTMS have reported a trend towards better treatment effects in depressed patients with the BDNF val/val genotype [31]. Furthermore, L/L homozygotes of the serotonin transporter-linked polymorphic region (5-HTTLPR) and C/C genotype of the serotonin 1A receptor (5-HT1A) have been identified as potential predictors of the response to rTMS [14, 32]. Thus, in the present study, a genetic analysis of BDNF, 5-HTTLPR, and 5-HT1A will be performed.

### Objectives, hypotheses and trial design

The present trial is a single-centre, parallel-group, superiority, double-blind randomized controlled trial examining the effect of once-a-day iTBS compared to sham iTBS for 10 consecutive workdays in outpatients with moderate, unipolar depression. The main goals are to evaluate the effectiveness of 2 weeks of iTBS over the dorsolateral left DLPFC for the reduction of depression symptoms and to identify variables associated with the variability in the effect of iTBS on depressive symptoms. The main hypotheses of the trial are as follows:

(1) Patients receiving iTBS will display significantly larger reductions in depressive symptoms measured by the Montgomery-Aasberg Depression Rating Scale (MADRS) [33] and Becks Depression Inventory II (BDI-II) [34] compared to patients receiving sham stimulation. (2) Reduction in depressive symptoms will be significantly associated with a concomitant improvement in executive functions measured by neuropsychological tests. (3) Stronger connectivity at baseline between the DLFPC and the anterior cingulate cortices will be associated with better response to iTBS. (4) Variability in genetic measures will be significantly associated with treatment response to iTBS. (5) Variability in white matter structural measures of the brain will be significantly associated with the anti-depressive response to iTBS. The hypotheses will be investigated in separate work packages and will be reported in separate scientific articles.

## Methods

We used the SPIRIT reporting guidelines [35], see the attached SPIRIT checklist.

### Recruitment and study setting

Participants will be recruited prospectively, and the study will be performed at laboratories at the UIT, the Artic University of Norway, and the University Hospital of North Norway-Health Trust (UNN-HF).

Information about the research project is distributed at the outpatient clinic at the UNN-HF and to general practitioners in the city of Tromsø, Norway. Participants must contact the study themselves.

### Eligibility criteria

Participants must be between 22 and 65 years of age at inclusion, be fluent in the Norwegian language, and meet the diagnostic criteria of at least moderate depression according to the MADRS. A MADRS score ≥ 20 usually indicates moderate depression [33]. Patients must provide informed written consent, be able to follow the treatment schedule, and have a satisfactory safety screening for iTBS and MRI.

Patients will be excluded from the study if the clinical assessments performed at baseline indicate that the current depressive episode is in the mild range or, on the contrary, that the current episode fulfils the criteria for severe depressive episode requiring inpatient treatment, acute suicide risk is present or if the current depressive episode is clearly triggered by grief or a recent major stressful life event. The clinical assessment indicates the likelihood of bipolar disorder, dysthymic disorder, psychotic symptoms, alcohol or substance abuse/addiction in the last 6 months, current eating disorders, obsessive–compulsive disorders or posttraumatic stress disorder. Further exclusion criteria due to safety issues are a medical history of seizure, neurological or neurosurgical pathologies, cardiac or systemic disease, metallic prosthetic material or foreign objects (pacemakers, prosthetic eye equipment, etc.) and being pregnant or breast-feeding [36, 37]. Additionally, if patients have a previously diagnosed developmental disorder (learning disabilities, AD/HD or autism spectrum disorder) and a borderline personality disorder or is an active user of illegal drugs, they are also excluded.

No treatments are prohibited during the trial, but drug therapy must have been stable for the last 3 weeks prior to the first treatment day with iTBS and must be kept stable throughout the study until 4 weeks after the last day of iTBS treatment. All concurrent treatments will be recorded.

### Schedule of enrolment, interventions and assessments

Prior to enrolment, detailed information about the study is provided, and a medical doctor does an eligibility screening (safety check, inclusion/exclusion criteria) by telephone, and the consent form is sent by mail. If an eligible patient still wishes to participate after this step, the written informed consent form must be handed in at the study site prior to the baseline assessments. At the study site, a certified clinician (medical doctor or specialist in clinical psychology) performs a relatively brief clinical assessment. The participant thereafter fills out questionnaires and completes a cognitive assessment. Cerebral MRI and blood sample collection are conducted at the hospital laboratories. Preferably within 3 days and not more than a week after the baseline assessments, patients are allocated to receive either sham or active iTBS once a day for 10 consecutive workdays. A clinician blinded to the stimulation parameters will evaluate the level of depression after the 5th session of active iTBS/sham stimulation, after the 10th session of iTBS/sham stimulation and 4 weeks after the 10th stimulation. Cognitive assessment and questionnaires from the baseline assessment will be repeated after 10 stimulations. A final telephone assessment of depression level is done at close-out, 4 weeks after the last stimulation day. At close-out, group allocation is disclosed to the patient. If the patient still fulfils the criteria for moderate depression at this point, 10 once-a-day sessions with open-label iTBS are offered. Table 1 shows the *Schedule of enrolment, interventions and assessments.*

**Table 1:** Schedule of enrolment, interventions and assessments

Time point	Study period				
	Enrolment		Post allocation		Close-out
	Pre-enrolment	Baseline	After 5 stimulations with iTBS or sham	After 10 stimulations with iTBS or sham	4-week follow-up post-last stimulation day
Screening for eligibility and safety^a^	X	X			
Randomization	X				
**Allocation**		X			
Written informed consent		X			
**Clinical procedures**					
Demographics		X			
Brief medical history		X			
M.I.N.I		X			
Maudsley staging method		X			
Neuroimaging		X			
Blood sample		X			
MADRS		X	X	X	X
**Disclosure of allocation**					X
**Questionnaires**					
Beck Depression Inventory-II		X		X	X
Beck Anxiety Inventory		X		X	X
Pittsburgh Sleep Quality Index		X		X	X
WHO- five Well-Being Index		X		X	X
WHO QOL BREF		X		X	X
BRIEF-A		X		X	X
**Cognitive assessment**		X		X	

*Abbreviations*: *M.I.N.I *Mini International Neuropsychiatric Interview, *WHO* World Health Organization, *QOL* Quality of life, *BREF* brief version, *MADRS* Montgomery and Asberg Depression Rating Scale, *BRIEF-A* Behavior Rating Inventory of Executive Functions—Adult version

^a^Additional safety checks are performed prior to each stimulation

The following variables are documented during the baseline visit only: sociodemographic (age, gender, laterality, years of education, professional and marital status) and medical history (length of illness, duration of current episode, number of previous episodes, psychiatric and addictive comorbidities, somatic history, treatments prescribed, degree of therapeutic resistance according to the Maudsley staging method (MSM) [38], family history of depression). Cerebral MRI (including structural and rs-fMRI) will be performed in a Siemens Biograph molecular MR system. A blood sample is required for genetic analysis of polymorphisms for BDNF, 5-HTTLPR and 5-HT1A.

The following variables are evaluated at both baseline and the end of iTBS or sham stimulation: intensity of depression symptoms according to the MADRS and the BDI-II [33, 34]. Anxiety will be assessed using the Beck Anxiety Inventory (BAI) [39]. Functional status, health-related quality of life and well-being will be measured with the World Health Organization Quality Of Life Brief Version(WHOQOL-BREF) [40] and the World Health Organization Well-Being Index [41]. Sleep will be measured with the Pittsburgh Sleep Quality Index [42]. Cognitive performance will be assessed using validated neuropsychological tests with published normative data. Subtests from the Wechsler Adult Intelligence Scale-IV [43] will be used for the estimation of general verbal capacity (similarities subtest) and working memory (digit span subtest). Subtests from the California Verbal Learning Test II short form will be used to measure verbal memory [44], and the Rey-Complex Figure Test will be used as a measure of visual memory [45]. Aspects of executive functions will be assessed with selected subtests from the Colour-Word Interference Test, the Verbal Fluency Test and the Trail Making Test, all from the Delis Kaplan Executive Functioning System [46], and with the Wisconsin Card Sorting Test [47]. A computerized N-back task [48] will be used to measure reaction time, attention and working memory. The Behaviour Rating Inventory of Executive Functions-Adult version (BRIEF-A) will be used to assess patient-reported dysexecutive problems in everyday life [49].

### Primary outcome variables

The primary outcome variable is the change in the level of depressive symptoms measured with the clinician-rated MADRS [33] from baseline to after 10 stimulations.

Change in depression symptoms will also be assessed with the self-assessment form BDI-II [34]. The main outcome will be reported as continuous changes in depression scores.

### Secondary outcome variables and covariates

Secondary outcome variables are changes in anxiety, executive functions and quality of life from baseline to after the 10th stimulation. Additionally, the stability of these measures at the last follow-up is secondary outcomes. Genetic and cerebral MRI results obtained at baseline are used only as covariates. Other covariates are participant characteristics (age, sex) and depression characteristics (treatment refractoriness, symptom severity).

### Randomization, stratification, allocation and blinding

The RCT will be run with an allocation ratio of 1:1 to the two groups (iTBS and sham). We expect a 1:1 ratio of males to females since the groups will be stratified by gender in the randomization. The randomization will be performed in MATLAB (https://se.mathworks.com/products/MATLAB.html), with the “randperm” command that performs randomization in four permuted blocks to groups and gender in a single operation. The a priori randomization procedure in MATLAB will be performed by a research assistant prior to the start of the study. This research assistant is not involved in the data collection.

The research assistant will produce numbered coloured envelopes (e.g. pink = female, blue = male), each containing a number indicating the type of coil to be used (e.g. 1 = active, 2 = sham). The staff member who performs the stimulation procedure will open the envelope. The staff members who provide iTBS will not assess the patient. Clinicians who assess the patients will not be aware of the treatment group. Thus, neither the clinician who assesses the patient nor the patient will know which treatment is provided. A study identification code will be allocated to each participant upon entry. Their name or national identity number will not appear in the research data collected. Four weeks after the last stimulation day, treatment allocation will be disclosed to the patient.

### Sample size calculation

A meta-analysis [1] reported a large effect size (Hedges’ *g*) of 1.0 favouring iTBS over sham stimulation for the reduction of depressive symptoms in major depression. Hence, using sample size calculations in GPower [50] with an estimate of a large effect size in a repeated analysis of variance (ANOVA) analysis, two groups, alpha = 0.05 and power = 0.9, the required sample size is 68, 34 in each group. The critical *F* value for the repeated analysis (pre-post) is 3.99. A study by Li et al. [11] reported a mean change after 10 treatment sessions in the iTBS group of − 42% and − 17% in the sham group and a standard deviation (SD) of 3.9. By assuming a mean baseline MADRS score of 22 in each group in the present study, the expected mean posttreatment MADRS score in each group will be 13 (active) and 18 (sham). With the two groups, alpha = 0.05, a pooled SD of 6, and power = 0.9, the required sample size is 46, 23 in each group to obtain a significant group difference in a two-tailed *t*-test. When using the same numbers for power, alpha and effect size, analyses of single means with ANCOVA-like analyses with fixed effects, main effects and interactions assuming 2 groups and 3 covariates will require group sizes of 43, e.g. 86 patients in total (critical *F* = 2.33) with equal group sizes. The analyses of repeated measures will be performed with linear mixed models (LMMs), as it manages random missing data better than repeated ANOVA. Otherwise, the sample size estimation is comparable to repeated ANOVAs due to similar residual covariance matrix assumptions [51]. There is limited information about drop-out rates for iTBS, but a meta-analysis on rTMS [52] found the mean drop-out rate to be between 7 and 8%. Thus, assuming a total drop-out of 10% in each group, the present study must recruit a total of 96 patients. Based on data from Blumberger et al. [4], we estimate a mean decrease of approximately 43% in depressive symptoms over a 2-week treatment course in those receiving iTBS. A 10-day treatment period has approximately 43% in depressive symptoms over a 2-week treatment course in those receiving iTBS. A 10-day treatment period has previously been used in RCTs, with significant differences between the iTBS condition and the sham/placebo condition [11, 53].

### ITBS interventions

Stimulation will be performed with a Mag & More PowerMag EEG 100 system (https://magandmore.com) with a double PMD70 p-cool (fluid-cooled) figure-eight coil. Sham stimulation will be performed with a double PMD70 p-cool figure-eight sham coil. The sham coil has an identical look, weight and sound compared to the true coil and delivers electrical stimulation that can be felt on the skin but without penetrating the skull.

A high-resolution T1-weighted image obtained at baseline will be used for real-time MRI-guided neuronavigation for optimal coil positioning. A Localite TMS-navigator system (https://www.localite.de) will be used for the determination of the target area [54] in MNI-space (*X* =  − 38, *Y* = 44, *Z* = 26) within the left DLPFC prior to the first day of stimulation. Additionally, we will use the Beam F3 method [55] for comparison with the stimulation point found by the neuronavigation [56]. The stimulation point on the scalp obtained by neuronavigation will be recorded with the coordinate system used in the Beam F3 method for use in stimulation sessions 2–10.

iTBS will be delivered with the same parameters as in Blumberger et al. [4] with stimulation intensity at 120% of resting motor threshold (RMT) triplet 50 Hz bursts repeated at 5 Hz; 2 s on and 8 s off, 600 pulses per session with a total duration of 3 min 9 s.

RMT is determined by observable right-handed thumb twitches (musculus abductor pollicis brevis) in three of five trials while delivering stimulation to the left primary motor cortex [57]. If a patient reports that stimulation intensity at 120% of RMT is intolerable at the first stimulation, the stimulation intensity will be titrated from 100% on day 1 to 120% on day 3. RMT will be determined prior to each stimulation, making sure not to stimulate above the suprathreshold intensity of 120% [58]. ITBS or sham stimulation will be provided once a day for 10 days for two consecutive weeks (except Saturdays and Sundays). Each patient will start treatment at approximately the same time between 9 am and 3 pm during the 10-day treatment period. All iTBS procedures, including RMT assessment, will be performed by trained personnel not blinded to the study condition. Participants will be monitored by study staff for 10 min after the stimulation and can thereafter leave the study site. The trial adheres to the latest safety recommendations for rTMS [36, 37].

A structured form will be used to document the side effects after each day of iTBS. Any side effects will be documented in the individual’s file. An analysis of side effects will be reported in the publication.

The criteria for discontinuing or modifying allocated interventions for a given participant include the following: worsening of disease, report of adverse side effects, withdrawal of consent, change in drugs or not receiving stimulation on more than 2 days. If the patient is absent from one or two stimulations, these will be added to the treatment schedule. For safety reasons, participants will be asked about sleep, alcohol, illegal drugs and medication use prior to each simulation. Participants will be asked about their well-being prior to and after each iTBS stimulation. The participants will be asked to drink no more than one unit of alcohol the night before stimulation. If the patient reports drinking more alcohol, stimulation will be postponed to the day after. Stimulation will also be postponed if the participant reports significant tiredness and/or lack of sleep the night before.

### Statistical analysis plan

Descriptive analyses of the data collected during each patient’s evaluation will be conducted up until the final evaluation. The time points for analyses are after the 5th iTBS session, 10th iTBS session and 4 weeks after the last stimulation. The follow-up 4 weeks after is used to test the stability of the reduction in depressive symptoms after iTBS and the change in symptoms after sham treatment. The effect of the intervention will be assessed as the linear change in depression scores measured on the MADRS and BDI-II.

Single means (e.g. baseline data) will be analysed with statistical methods within the framework of general linear models (GLMs) (ANOVA, linear regression, Pearson correlation, *t*-tests) if the statistical assumptions for GLMs are met. In the case of nonnormal data, nonparametric statistics will be performed. Changes in quantitative data over time will be analysed by linear mixed models (LMM), which allow for a combination of fixed and random effects. LMM estimates the parameters using all available patient data, is thus less sensitive to missing data, and has flexible modelling options for the residual covariance structure of the data [49].

Analyses of brain imaging data will be performed in separate programs according to the type of data. FreeSurfer (FS) [59] (https://surfer.nmr.mgh.harvard.edu/) will be used for the preprocessing of structural imaging data, and permutation analysis of linear models (PALM) will be used to perform statistics on structural data preprocessed in FS and in analyses where imaging data are combined with other data as covariates or factors [60]. Statistical Parametric Mapping 12 (https://www.fil.ion.ucl.ac.uk/spm/software/spm12/) will be used for the preprocessing of functional MRI data, and GIFT (https://www.nitrc.org/projects/gift) will be used for independent component analyses (ICA) and statistical analyses of rs- fMRI.

Missing data will be described for each group at all time points of measurement, and imbalances will be evaluated. Missing data will not be imputed in the analyses. Any patient who drops out will be described according to group, exit date, exit reason, characteristics at inclusion and last data collected.

An alpha value of 0.05 will be used for all analyses, and Bonferroni corrections will be applied when appropriate for controlling the type 1 error rate.

### Data management plan

The data management plan for this project was reviewed and approved by the Regional Committee for Medical Research Ethics Northern Norway (REC), UIT and UNN-HF. Two senior researchers from the UIT, but not affiliated with this trial, will be appointed as study monitors to oversee documentation of informed consent for all participants and verify that the data management plan is followed. All data will be stored on secure network locations governed by the university hospital. The data management plan for this project was reviewed and approved by REC, UIT and UNN-HF.

## Discussion

The effect of iTBS on depressive symptoms is generally well documented even if the number of RCTs is low, but less is known about the factors contributing to the variability in the response [1, 6, 12]. The present RCT will therefore include measures that previously have been proposed to be relevant for the effect of iTBS in a relatively large sample compared to most previous RCTs and clinical trials [6, 10]. The inclusion of measures from various sources such as brain imaging, genetics and neuropsychological assessment will provide the opportunity to identify variables associated with a favourable therapeutic response to iTBS but also for reduced or absent responses. iTBS trials have mostly included patients with treatment-resistant depression [6, 10]; however, the present trial welcomes both treatment-naïve and treatment-resistant patients, which may allow us to gain knowledge about iTBS as a first-line treatment.

## Patient and public involvement

The research group collaborates with the organization Mental Health Norway (https://mentalhelse.no/om-oss). Meetings have been held with a representative on the consent form, other study-related advertisements, procedures and recruitment. Patients were not directly involved in the design of the trial. After the results are analysed, we will discuss the dissemination of results with the representative.

## Trial status

Recruiting. First enrolment: June 4, 2022. Protocol version 2 (the first version was submitted to the funding agency on August 31, 2020). Recruitment will be completed in December 2024.

### Supplementary Information

**Additional file 1.** Reporting checklist for protocol of a clinical trial. 

## Data Availability

All publications will be open access as required by the funding agency. The data management plan for this project was reviewed and approved by REC, UIT and UNN-HF. The complete data file with anonymized data will be stored at the UIT open data repository (https://dataverse.no) after the publications from this trial are published. All investigators will have access to the final trial dataset. Authorship will follow the Vancouver guidelines.

## Abbreviations

iTBS: Intermittent theta burst stimulation
HF rTMS: High-frequency repetitive transcranial magnet stimulation
DLPFC: Dorsolateral prefrontal cortex
ACC: Anterior cingulate cortex
RMT: Resting motor threshold
MDD: Major depressive disorder
MRI: Magnetic resonance imaging
rs fMRI: Resting-state functional magnetic resonance imaging
FA: Fractional anisotropy
BDNF: Brain-derived neurotrophic factor
RCT: Randomized controlled trial
MADRS: Montgomery Aasberg Depression Rating Scale
BDI-II: Beck Depression Inventory II
MSM: Maudsley staging method
BAI: Beck Anxiety Inventory
LMM: Linear mixed model
ANOVA: Analysis of variance
ANCOVA: Analysis of covariance
GLM: General linear model
GIFT: Group ICA (Independent Component Analyses) of FMRI Toolbox
MNI: Montreal Neurological Institute
M.I.N.I.: Mini-International Neuropsychiatric Interview
5-HTTLPR: Serotonin-(5-hydroxy tryptamin)-transporter-linked polymorph region
5-HT1A: 5-Hydroxy tryptamin1A
UIT: University of Tromsø – The Arctic University of Norway
UNN-HF: University Hospital of North Norway – Health Trust
AD/HD: Attention deficit/hyperactivity disorder
SPIRIT: Standard Protocol Items: Recommendations for Interventional Trials
BRIEF-A: Behavior Rating Inventory of Executive Functions – Adult version
WHOQOL-BREF: World Health Organization Quality of Life Brief Version
REC: Regional Committee for Medical Research Ethics Northern Norway
PALM: Permutation analysis of linear models
RMT: Resting motor threshold
FS: FreeSurfer
ICA: Independent component analysis
